# Characterization of the Annonaceous acetogenin, annonacinone, a natural product inhibitor of plasminogen activator inhibitor-1

**DOI:** 10.1038/srep36462

**Published:** 2016-11-23

**Authors:** Stéphane Pautus, Mouad Alami, Fréderic Adam, Guillaume Bernadat, Daniel A. Lawrence, Allan De Carvalho, Gilles Ferry, Alain Rupin, Abdallah Hamze, Pierre Champy, Natacha Bonneau, Philippe Gloanec, Jean-Louis Peglion, Jean-Daniel Brion, Elsa P. Bianchini, Delphine Borgel

**Affiliations:** 1 Université Paris-Sud, INSERM UMR-S1176, 94276 Le Kremlin-Bicêtre, France; 2 Servier Research Institute, 11 rue des Moulineaux 92150 Suresnes, France; 3 Université Paris-Sud, BioCIS, 5 rue Jean-Baptiste Clément 92290 Châtenay-Malabry, France; 4 Department of Internal Medicine, Division of Cardiovascular Medicine, University of Michigan Medical School, Ann Arbor, MI 48109, USA; 5 Laboratoire de Pharmacognosie, BioCIS, Univ. Paris-Sud, CNRS, Université Paris-Saclay, UFR Pharmacie, 5 rue Jean-Baptiste Clément, 92290, Châtenay-Malabry, France; 6 AP-HP, Hôpital Necker, Service d’Hématologie Biologique, 75015 Paris, France

## Abstract

Plasminogen activator inhibitor-1 (PAI-1) is the main inhibitor of the tissue type and urokinase type plasminogen activators. High levels of PAI-1 are correlated with an increased risk of thrombotic events and several other pathologies. Despite several compounds with *in vitro* activity being developed, none of them are currently in clinical use. In this study, we evaluated a novel PAI-1 inhibitor, annonacinone, a natural product from the Annonaceous acetogenins group. Annonacinone was identified in a chromogenic screening assay and was more potent than tiplaxtinin. Annonacinone showed high potency *ex vivo* on thromboelastography and was able to potentiate the thrombolytic effect of tPA *in vivo* in a murine model. SDS-PAGE showed that annonacinone inhibited formation of PAI-1/tPA complex via enhancement of the substrate pathway. Mutagenesis and molecular dynamics allowed us to identify annonacinone binding site close to helix D and E and *β*-sheets 2A.

Plasminogen activator inhibitor type 1 (PAI-1) is a member of the serine protease inhibitor (serpin) superfamily. Proteins belonging to this family share a common structure including three *β*-sheets (A, B and C), nine α-helices (hA-hI) and a flexible reactive center loop (RCL). PAI-1 is a key regulator of the fibrinolysis system and the main inhibitor of tissue plasminogen activator (tPA) and urokinase plasminogen activator (uPA). High plasma levels of PAI-1 are related to the development of thrombosis as well as several other pathologies such as cardiovascular diseases and metabolic disturbances^1^,^2^,^3^. Moreover PAI-1 is able to promote tumor angiogenesis and high PAI-1 level in solid tumors are associated with a poor prognosis^4^,^5^. Therefore, development of small molecule PAI-1 inhibitors should prove useful not only in the treatment of thrombotic disorders but also in diverse disease states. PAI-1 inhibits its target proteases using the classical serpin mechanism. The RCL inserts into the active site of the target protease forming a 1:1 reversible Michaelis complex. The RCL is then cleaved by the protease resulting in an acyl-enzyme intermediate with covalent binding between the P1 residue of the RCL and the protease. Then, either PAI-1 undergoes the stressed to relaxed transition in which the *N*-terminal side of the RCL inserts into *β*-sheet A dragging the protease on the opposite side of PAI-1^6^ or the RCL can be cleaved by the protease and inserted into *β*-sheet A to form a cleaved “substrate-like” form of PAI-1^7^. PAI-1 is also an unstable protein and converts rapidly, with a half-life of only 1–2 hours at 37 °C, into a more stable inactive latent conformation by inserting its RCL into the central *β*-sheet^8^. *In vivo*, PAI-1 circulates mainly bound to vitronectin which stabilizes it in its active conformation^9^. Small molecule PAI-1 inhibitors were first reported in 1996 by Charlton *et al.*^10^, then several other PAI-1 inhibitors were described, although none of them are currently in clinical use^1^,^11^,^12^. They display various mechanisms of action and binding sites. PAI-039 or tiplaxtinin, the most studied PAI-1 inhibitor so far, was inactive against vitronectin-bound PAI-1 but displayed strong potency in animal disease models^13^. Tiplaxtinin worked by blocking covalent complex formation of PAI-1 with proteases and converting PAI-1 into a substrate. Docking studies together with mutagenesis localized its binding site in the region of helices D and E close to the vitronectin binding site^14^. Three crystal structures of latent PAI-1 cocrystallized with compound AZ3976^15^ and active PAI-1 cocrystallized with compound CDE-096^16^ and Embelin^17^ were also reported. They show different binding site on PAI-1 and different mechanism of action. AZ3976 and Embelin bind to the flexible joint region between *α*-helix D and *β*-strand 2A. AZ3976 accelerates the latency transition of active PAI-1, while Embelin converts PAI-1 into a substrate for its target proteases. CDE-096 binds to a pocket close to the RCL composed of residues from *β*-sheets B, *β*-sheets C and *α*-helix H and prevents formation of the Michaelis complex with the proteases. Here we report the PAI-1 inhibitory activity of annonacinone, a natural aliphatic polyketide of the Annonaceous acetogenins group. We demonstrate that annonacinone displays higher potency than tiplaxtinin *in vitro* and *ex vivo* on human plasma and then investigate its mechanism of action and binding site.

## Results

### Identification of a novel PAI-1 inhibitor, annonacinone

In order to identify new compounds inhibiting the effect of PAI-1, a high throughput screening assay was set up, and used to screen our compounds library (around 4000 molecules). Compounds were tested for their ability to reduce the rate of tPA inhibition by PAI-I in a continuous chromogenic assay. Tiplaxtinin, a chemical compound known to inhibit PAI-I activity^13^ was used as a positive control. Addition of tiplaxtinin at 50 μM reduced the apparent rate constant of PAI-1 inhibition from 4.4 ± 0.3 × 10^−3^ s^−1^ to 1.7 ± 0.3 × 10^−3^ s^−1^ (Table 1). In the screening test, 37 compounds significantly reduced PAI-1 inhibition rate constant at 50 μM. Compounds were then tested at 10 μM, and 16 compounds still displayed potency. Among them, annonacinone, from our local collection BioCIS, was the most potent compound; it reduced the inhibition rate constant from 4.4 ± 0.3 × 10^−3^ s^−1^ to 6.4 ± 0.7 × 10^−4^ s^−1^ (see Supplementary Fig. S1A). IC_50_ were also measured in the chromogenic screening assay, annonacinone appeared to be more potent than tiplaxtinin (9 ± 1 μM and 28 ± 1 μM respectively, see Supplementary Fig. S1B). As a control, annonacinone was also tested on tPA-catalyzed substrate hydrolysis without PAI-1 and showed no effect.

A couple of annonacinone analogues belonging to the same group of compounds (annonacin, isoannonacin, rolliniastatin-2 and isorolliniastatin-2), available in our laboratory, were tested in the chromogenic screening assay. All four analogues have potency for PAI-1 inhibition (Table 1). Annonacin and isorolliniastatin-2 displayed similar potency to that of annonacinone (IC_50_ = 6 ± 3 μM, 10 ± 2 μM and 9 ± 1 μM respectively) while rolliniastatin-2 was similar to tiplaxtinin (IC_50_ = 19 ± 1 μM and 28 ± 1 μM respectively). Isoannonacin displayed only moderate potency (IC_50_ > 50 μM).

The selectivity of annonacinone for PAI-1 inhibition was also evaluated by measuring its effect on two of the main plasma serpins i.e. α1-antitrypsin and antithrombin toward their physiological targets, neutrophil elastase and factor Xa, respectively. In a continuous chromogenic assay, the apparent inhibition rate constant (k_app_) of neutrophil elastase by α1-antitrypsin previously incubated with 50 μM annonacinone (k_app_ = 1.1 × 10^−3^ s^−1^) was found similar to that measured with untreated α1-antitrypsin (k_app_ = 1.2 × 10^−3^ s^−1^). The same was also true for the fondaparinux-catalyzed inhibition of factor Xa by antithrombin pre-incubated with annonacinone or control buffer (k_app_ = 8.3 × 10^−3^ s^−1^, or 7.9 × 10^−3^ s^−1^, respectively, Supplementary Fig. S2). This indicates that inhibitory activity of annonicone seems specific for PAI-1, at least among the serpins tested.

### Effect of annonacinone in a human plasma assay

**Figure 1:**
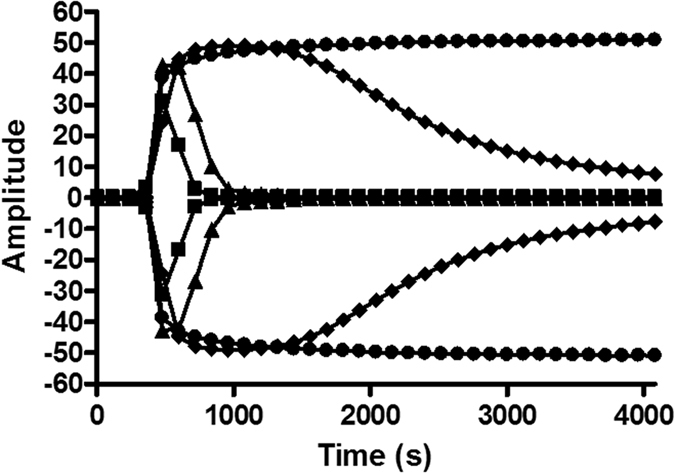
Effect of annonacinone and tiplaxtinin in thromboelastography on a plasma pool at 50 μM. Addition of tPA in plasma (closed square) resulted in quick lysis of the clot, further addition of PAI-1 resulted in complete inhibition of tPA fibrinolytic effect (closed dot). Addition of annonacinone at 50 μM (closed triangle) and tiplaxtinin at 50 μM (closed diamond) inhibited PAI-1 antifibrinolytic effect and reduced clot lysis time.

After demonstrating annonacinone activity *in vitro*, its effect in a plasma global coagulation assay, thromboelastography (TEG), was investigated. The most relevant parameters to study the effect of PAI-1 inhibition in TEG were the LY30 and the LY60, which measure the percentage of clot lysis 30 minutes and 60 minutes after the maximal amplitude (MA). Experiments conditions were set as addition of tPA in plasma resulted in quick lysis of the clot (LY60 = 96.8%), whereas further addition of PAI-1 completely inhibited the fibrinolytic effect of tPA (LY60 = 0%). Pre-incubation of PAI-1 with tiplaxtinin and annonacinone resulted in shortened lysis time. At 50 μM, LY60 was shortened by 50% in presence of tiplaxtinin while complete inhibition (LY60 = 94%) of the PAI-1 effect was observed with annonacinone (Fig. 1). No significant effects were observed on the other parameters (clotting time R, clot formation time K, angle, maximal amplitude MA) (see Supplementary Table S1). The LY60 values of three independent experiments were plotted against annonacinone concentration to calculate the IC_50_, 2.3 ± 1 μM.

### Effect of annonacinone *in vivo* on thrombolysis efficiency induced by rtPA

**Figure 2:**
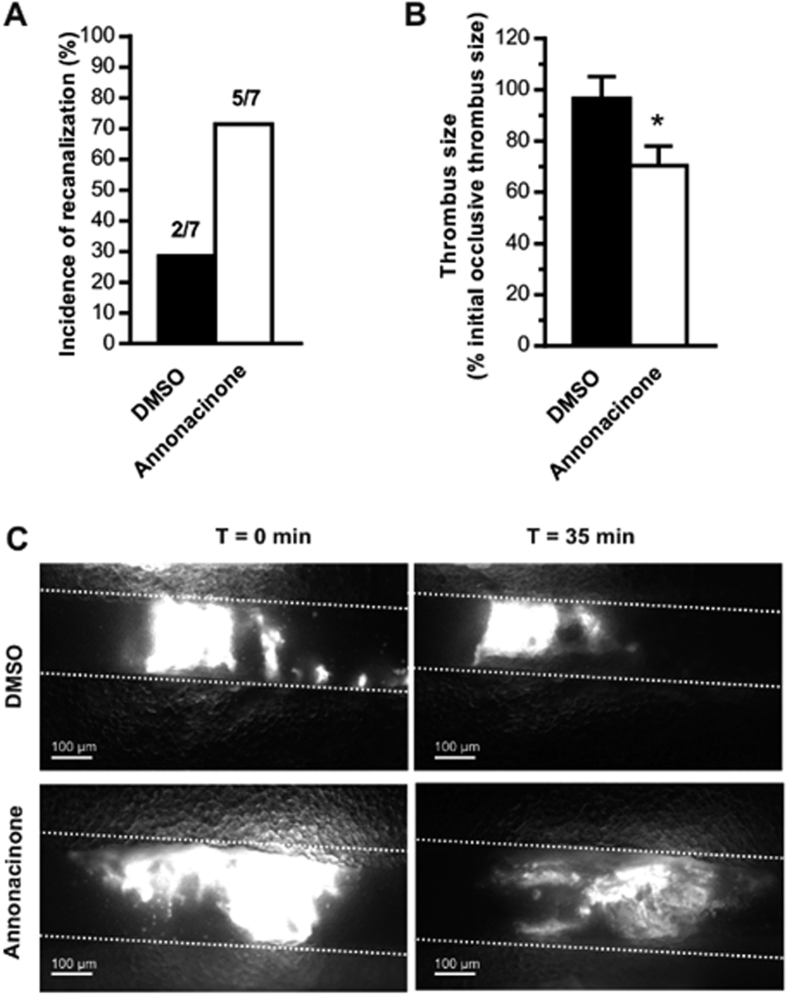
Effect of annonacinone *in vivo* on thrombolysis induced by rtPA. (**A**) Incidence of recanalization after 1 hour of treatment by the thrombolytic cocktail composed by rtPA (40 μM) and Argatroban (200 μM) supplemented by 0.2% DMSO (control condition) or 20 μM annonacinone. Numbers above the bars indicate the total number of vessels recanalized per group of 7 mice. (**B**) Quantification of thrombus surface after 35 minutes of treatments. Results are expressed as percentages of the thrombus surface compared to the initial thrombus size. n = 7 mice per condition. (**C**) Representative images of intravital microscopy showing thrombus 10 minutes after vessel occlusion (t = 0 min) and at 35 minutes after treatments (t = 35 min). Bar = 100 μm.

To further investigate whether, *in vivo,* annonacinone potentiates the thrombolytic effect of tPA, we tested its efficacy in a murine model of thrombolysis performed by intravital microscopy^18^. In this model, DMSO (0.2%, used as vehicle) or annonacinone (20 μM) were locally applied onto FeCl_3_-occluded venules in the presence of a low concentration of rtPA (Actilyse, 40 μM), but also in the presence of Argatroban (200 μM), a thrombin inhibitor. Indeed FeCl_3_-induced occlusive thrombosis has been shown to be highly dependent on thrombin^19^,^20^ and therefore, the thrombin activity at the site of endothelial injury could interfere with thrombolysis and mask the action of rtPA and annonacinone. The evaluation of the incidence of recanalization indicated that while 28% of mice exhibited a partial restoration of blood flow in presence of a low dose of rtPA and 0.2% DMSO, the addition of annonacinone to rtPA increased to 71% the recanalization (Fig. 2A). Moreover, evaluation of the thrombus size indicated a significant decreased of its initial size by 29.7 ± 7.7% (n = 7; p < 0.05) after 35 minutes of annonacinone treatment compared to DMSO (3.4 ± 8.5% of decreased) (Fig. 2B,C). Altogether, these results suggest that annonacinone potentiates the fibrinolytic action of tPA.

### Elucidating the mechanism of action of annonacinone

**Figure 3:**
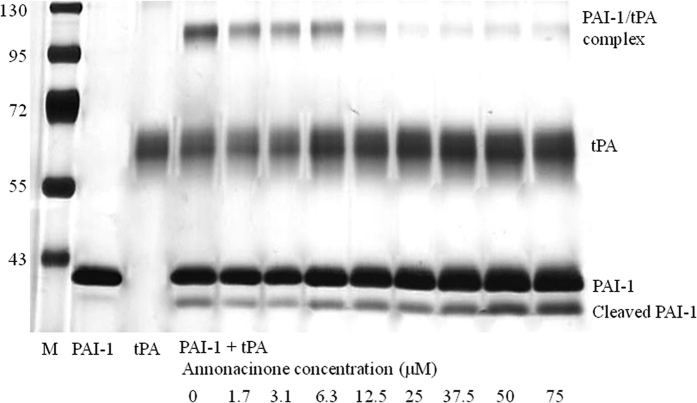
Elucidation of Annonacinone mechanism of action by SDS-PAGE. Influence of annonacinone on the PAI-1/tPA interaction on SDS-PAGE. PAI-1 (4 μL, 1 μM) was pre-incubated with 16 μL containing indicated concentrations of annonacinone (2% DMSO) for 30 min at 37 °C. Human tPA (4 μL, 0.5 μM) was then added to the mixture and incubated for 15 min at 37 °C. Reaction was stopped by addition of 4X SDS-PAGE loading buffer followed by immediate boiling. Samples were then analyzed on SDS-PAGE followed by silver staining. The margins indicate (left) molecular weights (×10^3^ Da) of the markers (M), (right) the migration positions of relevant species, and (bottom) the experimental conditions.

The influence of annonacinone on PAI-1/tPA covalent complex formation was also assessed on SDS-PAGE. A concentration-dependent inhibition of PAI-1/tPA complex formation was observed in the presence of annonacinone (Fig. 3). The increase of cleaved PAI-1 and free tPA levels indicates that annonacinone inhibits PAI-1 dependant tPA inhibition probably by interfering with RCL insertion into β-sheet A and thus favouring the reaction between PAI-1 and tPA to proceed through the substrate pathway. Annonacinone also induced a dose-dependent enhancement of the intensity of free PAI-1 that may also result from allosteric modification of PAI-1 impairing its reaction with tPA.

One of the possible mechanisms for annonacinone PAI-1 inhibition is to accelerate the transition from active PAI-1 to latent inactive PAI-1. In order to measure this effect, the elastase mediated cleavage of PAI-1 was used. It is well known that elastase is able to cleave the RCL of active PAI-1 between residues P4 and P3^21^. However, in the latent form, the PAI-1 RCL is buried in *β*-sheet A and is thus not accessible for cleavage by elastase. Consequently, compounds enhancing PAI-1 latency should show a decrease in the cleaved PAI-1 band on SDS-PAGE. No effects on the latency transition of PAI-1 were observed with annonacinone up to 50 μM (see Supplementary Fig. S3A,B).

It was shown that, as for many other serpins, PAI-1 RCL can insert into another PAI-1 molecule to form polymers^22^. The effect of annonacinone on PAI-1 polymerization was investigated using native PAGE as previously reported^23^. Incubation with increasing concentrations of annonacinone did not permit to show formation of PAI-1 polymers but resulted in an extinction of the band corresponding to native PAI-1 (Supplementary Fig. S3C). The diffuse migration of PAI-1 may resulted from high molecular weight polymers or PAI-1 aggregates formation as same samples on SDS-PAGE showed no diminution of PAI-1 band (Supplementary Fig. S3D).

### Effect of annonacinone in presence of vitronectin

**Figure 4:**
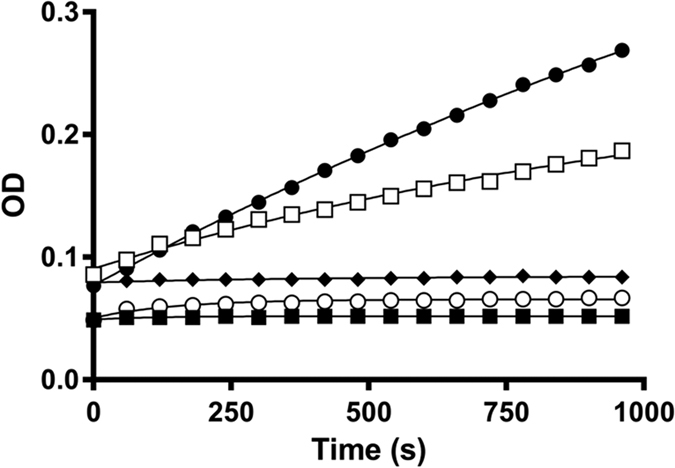
Effect of annonacinone on the tPA-catalyzed spectrozyme^®^ tPA hydrolysis in presence of PAI-1 and vitronectin (VN). Hydrolysis of the chromogenic substrate by tPA (closed dot). Addition of PAI-1 (opened dot) inhibits tPA substrate hydrolysis (k_app_ = 1.31 ± 0.16 × 10^−2^ s^−1^). Addition of PAI-1 and VN (closed square) also resulted in tPA inhibition (k_app_ = 1.05 ± 0.28 × 10^−2^ s^−1^). Pre-incubation of 50 μM annonacinone with PAI-1 for 20 min at 37 °C and further addition of VN for another 20 min at 37 °C (opened square) restored tPA substrate hydrolysis (k_app_ = 2.39 ± 0.32 × 10^−3^ s^−1^). On the other hand, annonacinone was inactive at 50 μM when added to a mixture of PAI-1 pre-incubated with VN for 20 min at 37 °C (closed diamond, k_app_ = 2.16 ± 0.17 × 10^−2^ s^−1^).

The effect of annonacinone on PAI-1/vitronectin complex was investigated by adding vitronectin (VN) in the chromogenic kinetic assay before and after pre-incubation of PAI-1 with annonacinone. When PAI-1 is pre-incubated with vitronectin, further addition of 50 μM annonacinone or DMSO 1% resulted in similar apparent rate constant (2.16 ± 0.17 × 10^−2^ s^−1^ and 1.05 ± 0.28 × 10^−2^ s^−1^ respectively) showing that annonacinone is inactive on PAI-1 bound to vitronectin as previously observed with tiplaxtinin^24^. On the other hand, when annonacinone is pre-incubated with PAI-1, it completely inhibit binding of PAI-1 to vitronectin as the IC_50_ obtained was similar from that without vitronectin (10 ± 2 μM and 9 ± 1 μM respectively, Fig. 4).

### Identification of annonacinone binding site

Site directed mutagenesis was used to investigate annonacinone binding site on PAI-1. The activity of annonacinone on five mutants (Y79A, T94A, D95A, R118A and K122A) belonging to the hydrophobic pocket between s2A, hD and hE close to vitronectin binding site was measured and compared to that of PAI-1 WT. These mutants were chosen for their ability to interact strongly with small molecule by hydrogen bonding and/or hydrophobic interactions. Results obtained undoubtedly confirm binding of annnonacinone in this site (Table 2). The dramatic decrease of annonacinone activity observed with T94A and Y79A (42 ± 6% and 12 ± 2% of WT activity respectively), clearly indicates strong interaction of annonacinone with these two residues. On the other hand, mutation of R118 and D95 to an alanine, increase annonacinone activity (264 ± 40% and 163 ± 24% of WT activity respectively) showing unfavourable interaction of annonacinone with these residues. Alanine mutation of surface loop residue K122 showed no effect.

### Docking and molecular dynamics

**Figure 5:**
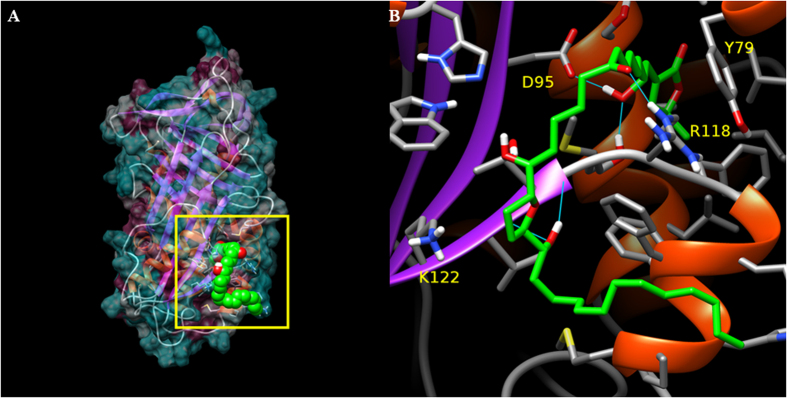
Depiction of a putative binding mode of annonacinone against the active form of PAI-1. (**A**) Overall view of the complex, with the solvent accessible surface depicted as semi-transparent cyan when polar, and purple when nonpolar. (**B**) Detailed view of the binding site. Cyan lines indicate hydrogen bonds. The name of residues identified by site directed mutagenesis is printed in yellow.

Overall examination of the molecular dynamics trajectory of the complex reveals that while annonacinone and PAI-1 remain bound to each other, the long aliphatic chain is the subject of large amplitude fluctuations from its position obtained by docking, and eventually seems to adhere to the hydrophobic surface of PAI-1 in the neighborhood of the vitronectin binding site (Fig. 5A) notably to the side chain of Phe114 as well as the non-terminal, hydrophobic part of that of Arg115. Investigation and comparison of the volume of the binding site in the presence and absence of annonacinone showed a lower average value when the ligand is bound (see Supplementary Fig. S5). At the same time, movement of the reactive loop was found of lower amplitude when the binding site is occupied by annonacinone (see Supplementary Fig. S6). A closer look at the reference ligand binding pocket shows that the α,β-unsaturated lactone head of annonacinone penetrates more deeply into it during the course of the simulation, progressively establishing several hydrogen bonds, notably with residues Asp95 and Arg118 (Fig. 5B), which were also identified by site-directed mutagenesis. Although the hydrogen bond is not visible in Fig. 5B, Lys122 residue participated a significant proportion of the time, as well as backbone atoms belonging to Ser119 and Val121.

## Discussion

In order to discover novel molecules inhibiting the effect of PAI-1, a screening of around 4000 molecules was performed. Unlike other previous screenings, mostly performed measuring residual tPA activity^14^,^25^,^26^,^27^, this screening measured the inhibition rate of tPA-catalyzed spectrozyme^®^ tPA hydrolysis in the presence of PAI-1. It allowed us to study the influence of compounds at early times and to, perhaps, identify compounds with different mechanism of action. During the screening, 37 molecules displaying potency for PAI-1 inhibition at 50 μM were identified and among them, annonacinone was the most potent. Annonacinone is a particularly interesting molecule as it is a natural product belonging to the Annonaceous acetogenins group specifically found in plants of the Annonaceae family. Indeed, these compounds are known as mitochondrial complex I inhibitors displaying a range of biological activities, including antitumoral and antiprotozoal properties^28^,^29^,^30^. Although they are present in fruits such as soursop and pawpaw, these compounds are also suspected of being environmental neurotoxins^31^. PAI-1 inhibition was also observed with four other acetogenins (annonacin, rolliniastatin-2, isoannonacin and isorolliniastatin-2), showing that several compounds of this family are potent PAI-1 inhibitors. Though synthesis and chemical modifications of Annonaceous acetogenins are very challenging^32^,^33^, it would be interesting to test more compounds belonging to that group.

Annonacinone was then tested *ex vivo* on pooled human plasma by thromboelastography (TEG). TEG was chosen as it yields data on the whole coagulation profile (clot lysis, kinetics and dynamics)^34^. The Influence of tPA and PAI-1 addition on healthy human plasma was already measured by TEG^35^ and TEG was also already used to investigate the effect of small molecule PAI-1 inhibitor on human plasma^36^. As previously observed in TEG^35^, tPA-treated plasma had short lysis time and further addition of PAI-1 increased lysis time to a normal healthy plasma level. Pre-incubation of PAI-1 with annonacinone completely inhibits PAI-1 anti-fibrinolytic activity of tPA-treated plasma in TEG and reduced clot lysis time more efficiently than tiplaxtinin. Besides its effect on fibrinolysis, annonacinone has no effect on coagulation factors involved in clot formation and on platelets, as it has no significant effect on the maximal amplitude (MA), which expresses clot strength and correlates to platelet function^37^ and on other parameters expressing kinetic of clot formation: reaction time (R), clot firmness (K) and the rate of clot formation (angle). Annonacinone potency was then confirmed *in vivo* using intravital microscopy, that allowed us to study the effect of this molecule on thrombolysis directly on the blood vessel of a living organism in real time, and is an appropriate technique to study antithrombotic agents^18^,^38^. In this mouse model, annonacinone was able to decrease thrombus size and to potentiate the thrombolytic effect of rtPA. These findings outline a new medicinal potential for Annonaceous acetogenins and evidence their influence in haemostasis, as persenone-A, an analogous polyketide from avocado (Lauraceae), also showed protective effects against arterial thrombosis *in vivo* and anti-platelet activity^39^. Several other small molecule PAI-1 inhibitors displayed *in vivo* efficacy^40^ but so far only three reached phase 1 clinical trial (tiplaxtinin, PAI-749 and PAZ-417) without further progression. In this context, the benefit-risk ratio of annonacinone deserves to be evaluated.

There are three main mechanisms for PAI-1 inhibition by a small molecule: conversion of active PAI-1 into an inactive latent form, prevention of Michaelis complex formation between PAI-1 and its target proteases, or inhibition of PAI-1/PAs complex formation via conversion of active PAI-1 in a cleaved substrate form^17^. SDS-PAGE analysis showed that annonacinone did not convert active PAI-1 into inactive latent PAI-1. Annonacinone inhibits PAI-1/tPA complex formation via enhancement of PAI-1 substrate behaviour. On the other hand, native PAGE analysis suggest that annonacinone enhance PAI-1 polymerisation or aggregation in a concentration dependant manner, this effect will inactivate PAI-1 and avoid formation of complex between PAI-1 and tPA. It seems that binding of annonacinone on PAI-1 will allosterically expose PAI-1 RCL and avoid withdrawal of the RCL into the central *β*-sheets. Then, the RCL will either be cleaved by tPA, to form substrate PAI-1, or may also insert in another PAI-1 molecule to form PAI-1 polymers. This finding is to be correlated with the lower amplitude of fluctuations observed during the molecular dynamics simulation, which could be the consequence of a propagation of the local deformation of the annonacinone binding site in the presence of this ligand. This mechanism is also coherent with previous studies suggesting that binding of negatively charged small molecules in the same pocket between s2A, hD and hE, induce substrate behaviour, followed by conversion of PAI-1 to inactive, polymeric forms^41^,^42^. PAI-1 RCL can insert in different positions (s7A, s4A and s1C) into another PAI-1 molecule to form different type of polymers^22^. Although, we cannot conclude about the annonacinone induced PAI-1 polymerization or aggregation, the elastase mediated cleavage experiment suggests that residues P4 and P3 of polymers RCL are still accessible for cleavage by elastase.

PAI-1 and vitronectin regulate each other function. Vitronectin is a physiological PAI-1 cofactor, which stabilizes PAI-1 in its active conformation, while binding of PAI-1 to vitronectin affects cell adhesion and motility^9^,^43^. The similar IC_50_ obtained with annonacinone with and without vitronectin (10 ± 2 μM and 9 ± 1 μM respectively) showed that annonacinone, when bound to PAI-1, prevents binding of vitronectin. However, experiments on the PAI-1/vitronectin complex showed that annonacinone loses its anti-PAI-1 activity when PAI-1 is bound to vitronectin. As PAI-1 exists mainly as a complex with vitronectin in plasma, the lack of effect of annonacinone against PAI-1/vitronectin complex should prevent the molecule of antithrombotic *in vivo* efficacy. Nevertheless, annonacinone *in vivo* efficacy was proven in our intravital microscopy experiment. Indeed, “free” PAI-1 exists in an active form after secretion from platelets, before it binds to vitronectin^44^ and other described PAI-1 inhibitors such as PAI-749 and tiplaxtinin, also devoid of activity against PAI-1 bound to vitronectin, display *in vivo* antithrombotic effect^1^,^23^.

Directed mutagenesis showed that annonacinone binds to the pocket between s2A, hD and hE similarly to other small molecule PAI-1 inhibitors such as tiplaxtinin, AZ3976 and embelin^14^,^15^,^17^. Molecular dynamics is in agreement with site directed mutagenesis results. Molecular dynamics showed that annonacinone binds to the pocket by its α,β-unsaturated lactone head using a network of hydrogen bonds with PAI-1 residues. On the other hand, annonacinone long aliphatic chain adheres to the hydrophobic surface outside of the pocket. Though it could not explain whether mutations have beneficial or detrimental effect on annonacinone/PAI-1 interaction, molecular dynamics definitely confirmed that PAI-1 residues Y79, T94, D95, R118 and K122 are involved with annonacinone binding. Annonacinone binding site is close to the binding region of vitronectin somatomedin B (SMB) domain^45^, binding of annonacinone will, thus, perturb PAI-1/vitronectin interaction. Indeed, molecular dynamics showed that binding of annonacinone in this pocket allosterically disturb PAI-1 interaction with vitronectin SMB and that annonacinone long aliphatic chain, outside of the pocket, will sterically prevent binding of SMB. Moreover, it was shown that vitronectin could interact, outside of its SMB domain, with a second site on PAI-1 between hD and hE^46^ that include annonacinone binding site. Annonacinone, share the same binding site and the same behaviour towards vitronectin bound PAI-1, than other PAI-1 inhibitors including tiplaxtinin and PAI-749^14^,^23^,^47^,^48^.

In conclusion, our work showed that, as well as their other biological properties, natural Annonaceous acetogenins, and particularly annonacinone, have an effect on fibrinolysis. Indeed, annonacinone is a potent inhibitor of PAI-1 *in vitro*, *ex vivo* and *in vivo*. Annonacinone mechanism of action and binding site on PAI-1 were also enlightened. Altogether, annonacinone appears to be a very promising antithrombotic agent and should be further studied.

## Materials and Methods

### Materials

Human recombinant PAI-1, human recombinant tPA single chain and human thrombin were purchased from Stago BNL (Asnières sur Seine, FR). Tiplaxtinin was obtained from Axon Medchem (Groningen, NL). Annonacin and annonacinone were isolated from the seeds of *Annona muricata* L. and rolliniastatin-2 was isolated from the seeds of *Annona squamosa* L., using previously described procedure^30^. Isoannonacin and isorolliniastatin-2 were obtained by semisynthesis under basic conditions, as described^49^. The substrate Spectrozyme-tPA was acquired from American Diagnostica (Stamford, CT, USA). Thromboelastography was performed on a Haemonetics TEG 5000. For *in vivo* studies, recombinant tissue-plasminogen activator (rtPA): Actilyse^®^ was from Boehringer (Ingelheim, Germany), Argatroban and Arganova^®^ from Mitsubishi Pharma Europe Ltd (London, UK), and Rhodamine 6G and ferric chloride from Sigma (St Louis, MO, USA). Buffer used for all experiments was PBS containing 20 mM sodium phosphate, 150 mM NaCl, 1 mM EDTA with 0.1% PEG-8000 and 0.1% BSA.

### High throughput screening

Compounds from our local collection BioCIS and from the Prestwick compound library (Prestwick chemicals, Illkirch, FR), a collection containing only FDA approved drugs (around 4000 compounds in total) were tested in a chromogenic assay measuring the inhibition rate of tPA-catalyzed spectrozyme^®^ tPA hydrolysis in the presence of PAI-1. Compounds (20 μL, final concentration 50 μM, 1% DMSO) were dispensed in a 96 well half area Greiner plate. A mixture containing 20 μL of PAI-1 (final concentration 20 nM) and 80 μL of Spectrozyme-tPA (final concentration 400 μM) was then added, and the mixture pre-incubated for 45 minutes at 37 °C. At the end of pre-incubation time, 90 μL of the mixture were added to another 96 well half area plate containing 10 μL tPA (final concentration 10 nM). The kinetic was then measured at 405 nm for 45 min at 37 °C in an Envision microplate reader. Progress curves were fitted using the pseudo first order equation to determine the apparent rate constant k.

### Thromboelastography

20 μL of CaCl_2_ (0.2 M), 10 μL of PAI-1 (final concentration 22 nM) and 10 μL of tested compounds were added in a TEG cup and pre-incubated for 20 min at room temperature. On the other hand, 1 mL of pooled citrated normal human plasma (pool of at least 10 healthy donors) was added to a vial containing 20 μL of Kaolin. 680 μl of the plasma/Kaolin mixture were then added to 20 μL of tPA (final concentration 9 nM). At the end of pre-incubation time, 320 μL of the plasma/tPA/kaolin mixture were transferred into the TEG cup and the thromboelastogram measured.

### Evaluation of the impact of annonacinone on thrombolysis efficiency induced by rtPA followed in real-time by intravital videomicroscopy

Thrombolysis was evaluated in an *in vivo* mouse model according to the method previously described^18^ with modifications. Briefly, platelets of anesthetized mice were fluorescently labeled *in vivo* by injection of rhodamine 6G (3.3 mg/kg) into the retro-orbital plexus, then thrombus formation was induced by topical deposition on the mesenteric vessels of ferric chloride solution (FeCl_3_; 15%) in 4- to 5-week-old mice. Thrombus growth was monitored in real-time with an inverted epifluorescent microscope (x10) (Nikon Eclipse TE2000U). Within the 10 minutes after total occlusion of the vessel, the effect of annonacinone was evaluated on the efficiency of rtPA-induced thrombolysis by applying directly on the occluded vessels a thrombolytic cocktail (40 μL) composed of 40 μM rtPA (Actilyse^®^), 200 μM Argatroban (Arganova^®^) and 0.2% DMSO or 20 μM annonacinone. The incidence of the recanalization, defined as a partial or complete restoration of flow associated to a decrease of more than 50% of the initial thrombus size, and the evolution of thrombus size were determined during 1 hour.

### Animal statement

Housing and experiments were done in accordance with French regulations and the experimental guidelines of the European Community. This project was approved by the local ethical committee CEEA 26 under the number APAFIS#2874-2015112512451547v2.

### PAI-1/tPA complex formation

PAI-1 (4 μL, 1 μM) was pre-incubated with 16 μL annonacinone (2% DMSO) for 30 min at 37 °C. Human tPA (4 μL, 0.5 μM) was then added to the mixture and incubated for 15 min at 37 °C. Reaction was stopped by addition of 4X SDS-PAGE loading buffer followed by immediate boiling. Samples were then analyzed on SDS-PAGE followed by silver staining.

### Elastase mediated cleavage of PAI-1

Selective cleavage of active PAI-1 by elastase was observed on SDS-PAGE. PAI-1 (5 μL, 2 μM) was incubated with or without compounds (200 μM, 5 μL, 5% DMSO) for 15 min at 37 °C. Elastase (5 μL, 1.4 μM) was then added and further incubated for 15 min at 37 °C. Reaction was stopped by addition of 4X loading buffer containing SDS and BME followed by immediate boiling. Samples were then analyzed on SDS-PAGE followed by silver staining.

### Native PAGE analysis of PAI-1

PAI-1 (1 μM) was mixed with various concentration of annonacinone or DMSO 1% for 30 min at 37 °C. Samples were mixed with 1/3 volume of native gel sample buffer or LDS sample buffer. The former were subjected to BN-PAGE and the latter were fractioned by SDS-PAGE. Proteins band were visualized by silver staining.

### Assay in presence of vitronectin

Compounds (20 μL) were dispensed in a 96 well half area Greiner plate. A mixture containing 20 μL of PAI-1 (final concentration 20 nM) and 40 μL of a chromogenic substrate (Spectrozyme-tPA, final concentration 400 μM) was then added, and the mixture pre-incubated for 20 minutes at 37 °C. 40 μL of vitronectin (final concentration 37.5 nM) was added and the mixture further pre-incubated for 20 min at 37 °C. At the end of pre-incubation time, 90 μL of the PAI-1/vitronectin/compound mixture were added to 10 μL of tPA (final concentration 10 nM) and the tPA catalysed substrate hydrolysis reaction was followed for 45 min at 405 nm. Progress curves were then fitted using the pseudo first order equation to determine the apparent rate constant k.

### Site directed mutagenesis

Mutants were constructed as previously described^50^. Based on computer modelling, the following mutants were prepared, T79A, T94A, D95A, R118A and K122A. The effect of annonacinone was measured at 50 μM for each mutant in our chromogenic screening assay. Progress curves were fitted using the pseudo first order equation to determine the apparent rate constant k. For each mutant, the ratio between the apparent rate constants with and without 50 μM annonacinone were calculated and then compared to that of PAI-1 WT to determine the % of annonacinone activity.

### Effect of annonacinone on α1-antitrypsin and antithrombin activities

5 μM human α1-antitrypsin (Sigma) or human antithrombin (Aclotin^®^, LFB) were incubated with 50 μM annonacinone (or 0.5% DMSO in reaction buffer as control) for 20 min at 37 °C. The mixtures containing α1-antitrypsin were diluted in N-Methoxysuccinyl-Ala-Ala-Pro-Val p-nitroanilide (Sigma) containing solution and substrate hydrolysis kinetic was triggered by addition of neutrophil elastase (Sigma) to a final volume of 100 μL containing 13 nM α1-antitrypsin, 130 nM annonacinone, 82 μM chromogenic substrate and 5 nM neutrophil elastase. The mixtures containing antithrombin were diluted in fondaparinux (Arixtra^®^, GlaxoSmithKline) and CS-11(22)-FXa (Hyphen Biomed) containing solution and substrate hydrolysis kinetic was triggered by addition of factor Xa (Stago BNL) to a final volume of 100 μL containing 51 nM antithrombin, 510 nM annonacinone, 200 μM chromogenic substrate, 5 μM fondaparinux and 2 nM factor Xa.

### Docking of annonacinone against PAI-1

X-ray structure of the active form of PAI-1 cocrystallized with 2,5-dihydroxy-3-undecylcyclohexa-2,5-diene-1,4-dione (accession code 3UT3) was retrieved from the Protein Data Bank. Coordinates for a low-energy conformation of annonacinone were generated using CORINA v3.44^51^. Pre-processing of the PDB structure as well as constraint-free molecular docking were performed using GOLD v5.1 software^52^ with the search efficiency parameter set to 200% and a binding site defined by a radius of 20 Å around the cocrystallized ligand. CHEMPLP with default parameters was used as an objective function^53^.

### MD simulation of the annonacinone–PAI-1 complex

Behaviour of PAI-1 and annonacinone in the presence of each other was further investigated by a molecular dynamics (MD) simulation starting from the highest-ranking pose yielded by the molecular docking protocol described above. Addition of the missing residues, as well as cancellation of the mutations present in the crystal structure of the protein were realized using Modeller v9.14 software package^54^. Simulation was performed with GROMACS v5.0.2 software package^55^ using AMBER99SB-ILDN force field^56^ in combination with TIP3P water model. A GAFF topology for the annonacinone ligand was generated using ACPYPE^57^. After solvation of the complex model in a periodic box with pre-equilibrated water molecules and addition of a sufficient number of sodium and chloride ions to reach electric neutrality with a 20 mM salt concentration, the system was subjected to two consecutive 1 ns MD simulations for equilibration purposes (the first one with Berendsen^58^ and the second one with Nosé-Hoover^59^ thermostat). Production MD simulation was then conducted with Nosé–Hoover thermostat and Parrinello–Rahman pressure coupling^60^ for a total simulated time of 50 ns. Atom coordinates were saved every simulated 20 ps for trajectory analysis. For comparison purposes, another molecular dynamics simulation was conducted under the same conditions as well as from the same initial protein conformation, but without annonacinone. The most representative conformation of the complex during the dynamics was identified after RMSD clustering of the trajectory performed using the GROMOS algorithm^61^ with a cut-off value of 1.5 Å. Volume of the binding site as a function of time with and without annonacinone was calculated using MDpocket^62^, using a frequency grid threshold isovalue of 0.35. RMS level of fluctuation of α-carbon atoms belonging to the reactive loop (defined as the Asn329–Glu350 subsequence) was calculated using standard GROMACS utilities.

## Additional Information

**How to cite this article**: Pautus, S. *et al.* Characterization of the Annonaceous acetogenin, annonacinone, a natural product inhibitor of plasminogen activator inhibitor-1. *Sci. Rep.* **6**, 36462; doi: 10.1038/srep36462 (2016).

**Publisher’s note:** Springer Nature remains neutral with regard to jurisdictional claims in published maps and institutional affiliations.

## Supplementary Material

Supplementary Information

## Figures and Tables

**Table 1:**
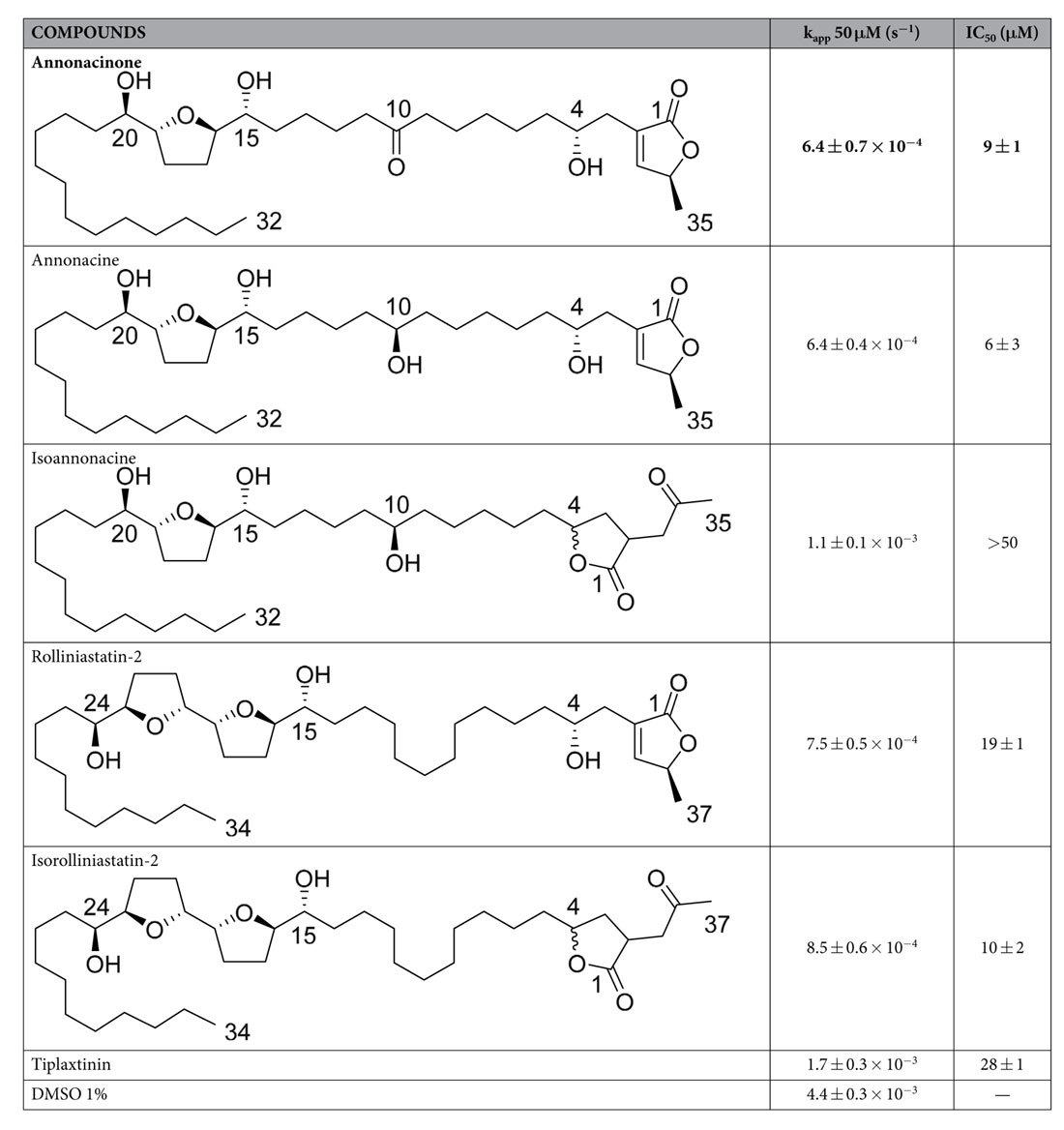
Structure and activity of annonacinone and others acetogenins in the chromogenic screening assay.

Each value represents the mean value ± SEM; n = 3.

**Table 2:** Inhibitory activity of 50 μM annonacinone with PAI-1 mutants in the screening chromogenic assay.

Mutation	Region	k_app_ DMSO 1% (s^−1^)	k_app_ Annonacinone 50 μM (s^−1^)	Annonacinone activity 50 μM (% WT)
WT	—	6,5 ± 0,6 × 10^−3^	9,6 ± 1,0 × 10^−4^	
Y79A	Helix D	4,0 ± 1,0 × 10^−3^	4,8 ± 0,9 × 10^−3^	12 ± 2
T94A	*β* -strand 2A	6,6 ± 0,9 × 10^−3^	2,3 ± 0,4 × 10^−3^	42 ± 6
D95A	*β* -strand 2A	1,1 ± 0,2 × 10^−3^	9,8 ± 1,5 × 10^−4^	163 ± 24
R118A	Surface loop	6,0 ± 0,9 × 10^−3^	3,4 ± 0,3 × 10^−4^	264 ± 40
K122A	Surface loop	1,9 ± 0,3 × 10^−3^	3,5 ± 0,9 × 10^−4^	80 ± 13

Curves were fitted using pseudo first order equation and the apparent rate constant was calculated for each mutant with and without 50 μM annonacinone. The ratio was then calculated and compared to that of PAI-1 WT (set as 100%) to determine the % of annonacinone activity at 50 μM for each mutant compared to WT. Each value represents the mean value ± SEM; n = 4.
