# Supplementary material for: Characterization of the Annonaceous acetogenin, annonacinone, a natural product inhibitor of plasminogen activator inhibitor-1
Source: Sci Rep. 2016 Nov 23;6:36462. doi: 10.1038/srep36462 (PMC5120274; doi:10.1038/srep36462)
Supplement: Supplementary Information [file srep36462-s1.pdf]

Characterization of the Annonaceous acetogenin, annonacinone, a natural product inhibitor of plasminogen activator inhibitor-1

Stéphane Pautus[1,2], Mouad Alami[3], Fréderic Adam[1], Guillaume Bernadat[3], Daniel A Lawrence[4], Allan De Carvalho[1], Gilles Ferry[2], Alain Rupin[2], Abdallah Hamze[3], Pierre Champy[5], Natacha Bonneau[5], Philippe Gloanec[2], Jean-Louis Peglion[2], Jean-Daniel Brion[3], Elsa P. Bianchini[1,7,*], Delphine Borgel[1,6,7]

Supplementary information

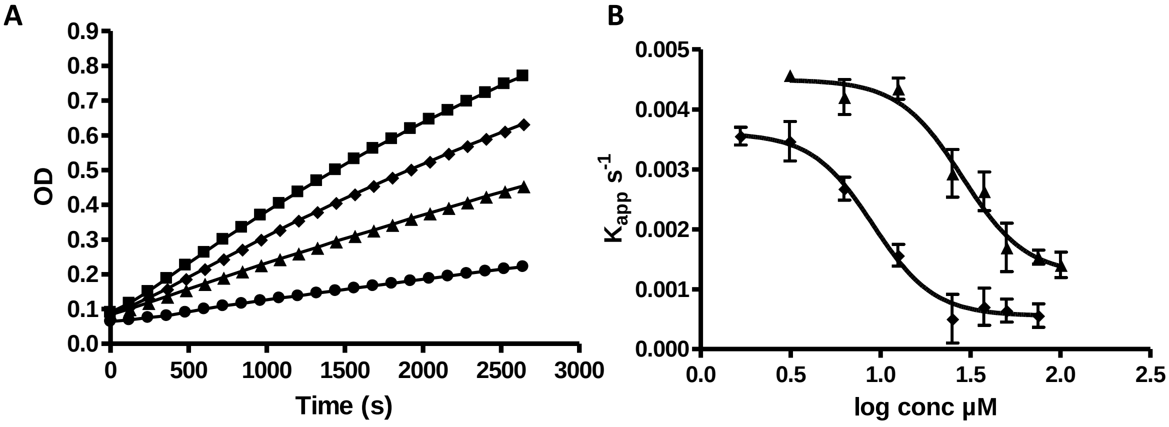

Supplementary Figure S1: (A) Effect of annonacinone and tiplaxtinin at 50 µM in the chromogenic screening assay. Hydrolysis of the chromogenic substrate by tPA (closed square). Addition of PAI-1 (closed dot) inhibits tPA substrate hydrolysis. Further addition of tiplaxtinin at 50 µM (closed triangle) and annonacinone at 50 µM (closed diamond) reduce the rate of tPA inhibition by PAI-1. Progress curves were fitted using the pseudo first order equation to determine the apparent rate constant k. (B) IC_{50} of annonacinone (closed diamond) and tiplaxtinin (closed triangle) in the chromogenic assay. The apparent rate constants were plotted against drug concentrations and fitted using Graphpad prism software (Graphpad software, La Jolla USA) to determine the IC_{50}

	R (min)	K (min)	Angle (°)	MA (mm)	LY30 (%)	LY60 (%)
tPA	5.9	-	69.8	15.7	94.3	96.8
tPA + PAI-1	6	2.3	72.7	23.1	0	0
tPA + PAI-1 + annonacinone 50 µM	6	1.6	73.5	21.4	87.1	93.6
tPA + PAI-1 + tiplaxtinin 50 µM	7	1.8	66.2	24.5	21.9	50.2

Supplementary Table S1: Clotting parameters of plasma treated with tPA 9 nM; tPA 9 nM + PAI-1 22 nM and tPA 9 nM + PAI-1 22 nM with annonacinone and tiplaxtinin on thromboelastography. Each value is the representative of three independent experiments.

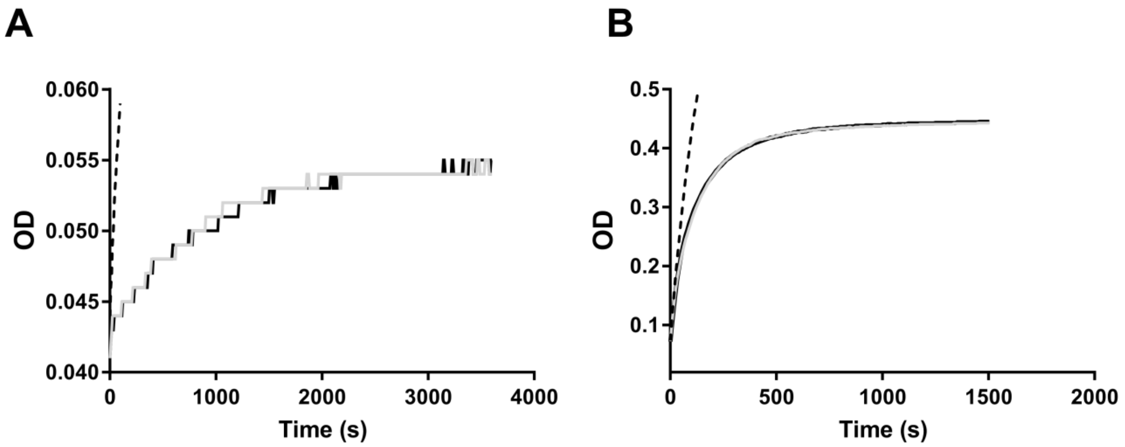

Supplementary figure S2: Effect of annonacinone on other serpins**.** A) Hydrolysis of specific chromogenic substrate by neutrophil elastase (dotted black line). Addition of 13 nM α1-antitrypsin (solid grey line) inhibits elastase substrate hydrolysis (k_{app}= 1.2 × 10^{-3} s^{-1}). Preincubation of 50 µM annonacinone with α1-antitrypsin for 20 min at 37°C (solid black line) resulted in a comparable inhibition rate of elastase by α1-antitrypsin (k_{app}= 1.1 × 10^{-3} s^{-1}). B) Hydrolysis of specific chromogenic substrate by factor Xa (dotted black line). Addition of 51 nM fondaparinux-bound antithrombin (solid grey line) inhibits factor Xa substrate hydrolysis (k_{app}= 7.9 × 10^{-3} s^{-1}). Preincubation of 50 µM annonacinone with antithrombin for 20 min at 37°C (solid black line) resulted in a comparable inhibition rate of factor Xa by fondaparinux-bound antithrombin (k_{app}= 8.3 × 10^{-3} s^{-1}).

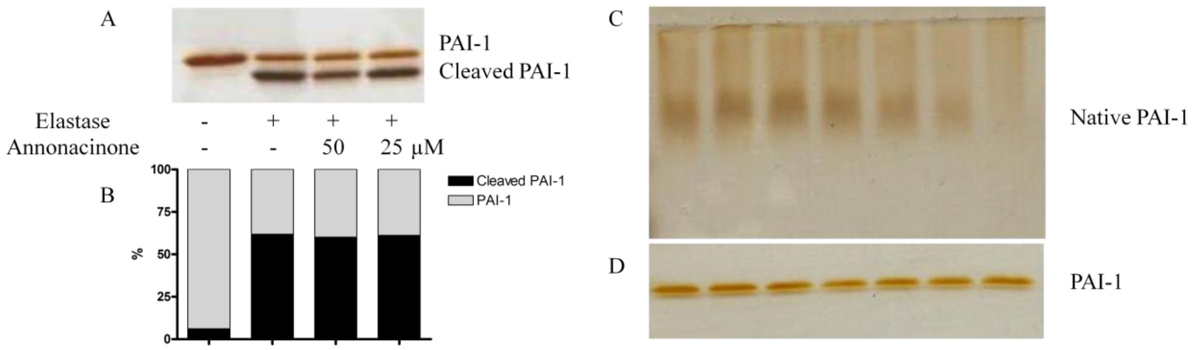

<u>Supplementary Figure S3</u>: A) Effect of annonacinone on elastase mediated cleavage of PAI-1 on SDS-PAGE. B) Band quantification using Scion Image Software (Scion Corporation, Frederick, MD, USA). C and D) Effect of annonacinone on PAI-1 polymerisation. PAI-1 (1 µM) was mixed with 0, 1.7, 3.1, 6.3, 12.5, 25, or 50 µM annonacinone (lane 1 to 7, respectively) and incubated for 30 min at 37 °C. The mixture was then mixed with native gel sample buffer and fractionated by native PAGE (C) or LDS sample buffer and fractionated on SDS-PAGE (D).

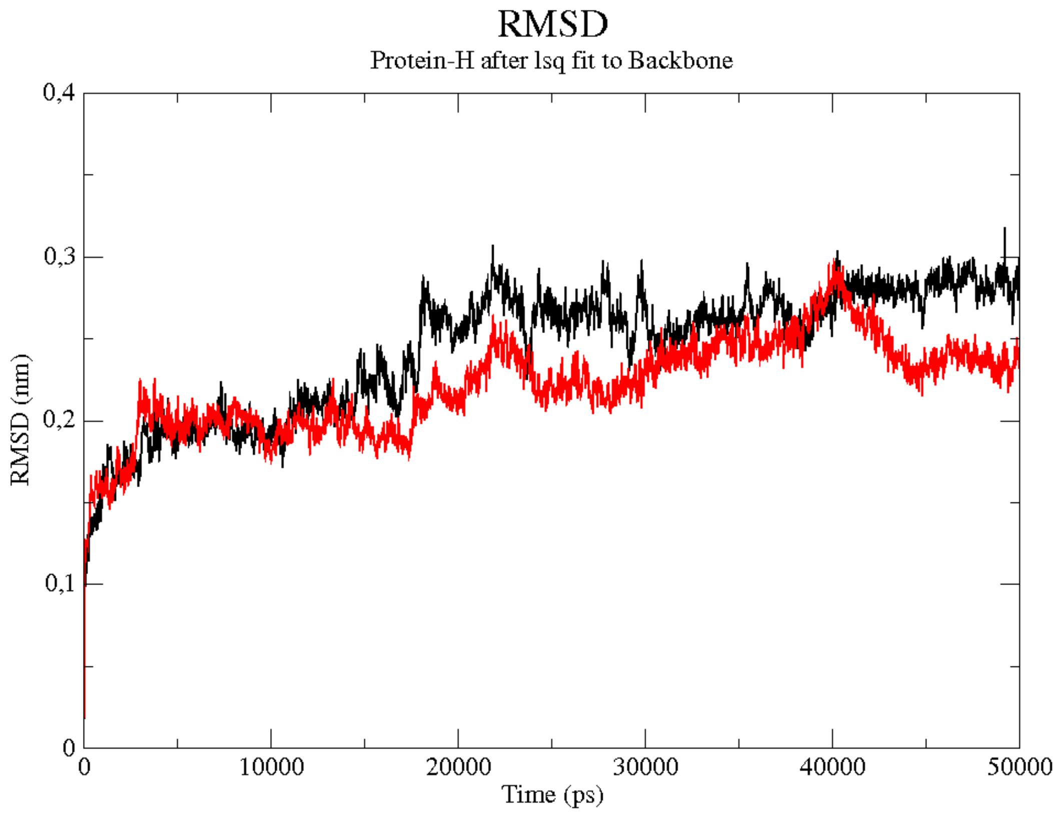

<u>Supplementary Figure S4</u>: Plots of PAI-1 RMSD as a function of time during molecular dynamics. (black: without annonacinone, red: with annonacinone.)

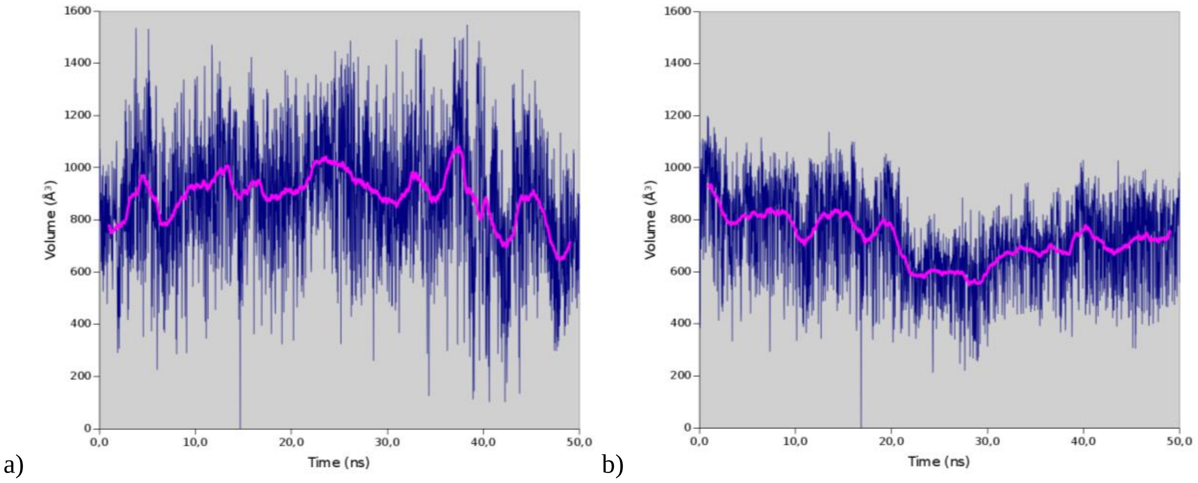

<u>Supplementary Figure S5</u>: Plots of annonacinone binding pocket volume as a function of time during molecular dynamics. (blue: actual value, pink: moving average, a) without annonacinone, b) with annonacinone.)

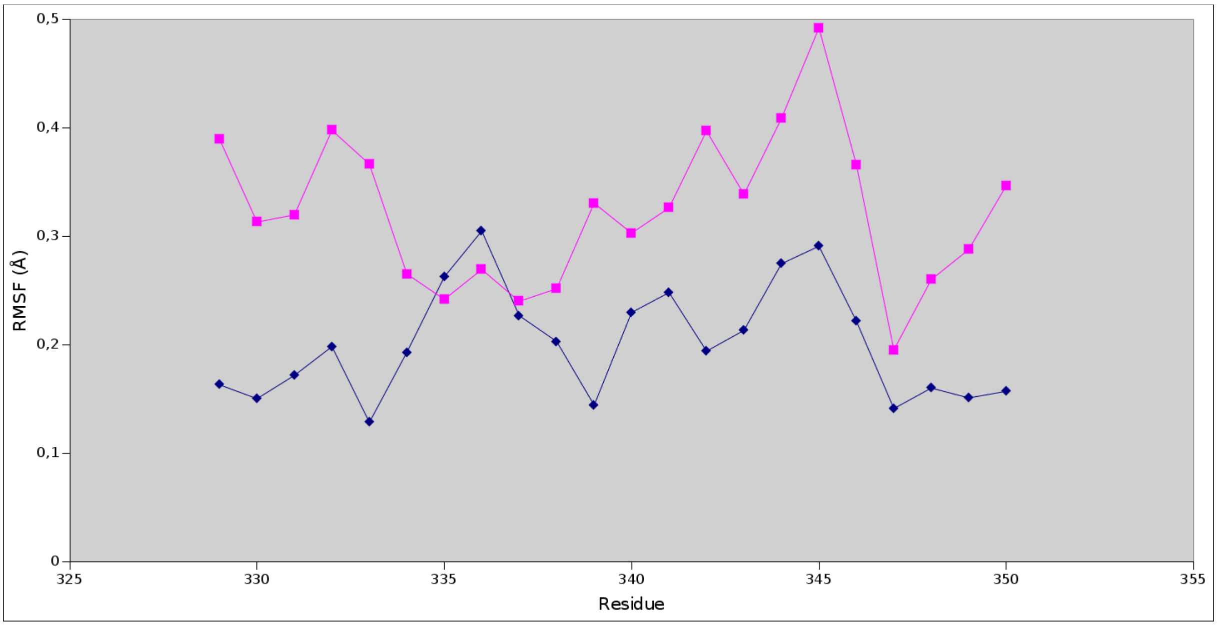

<u>Supplementary Figure S6</u>: Plots of RMS fluctuations of the reactive loop during molecular dynamics. (pink: without annonacinone, blue: with annonacinone.)
